# Appropriate Employment for Segregated Roma: Mechanisms in a Public–Private Partnership Project

**DOI:** 10.3390/ijerph17103588

**Published:** 2020-05-20

**Authors:** Lucia Bosakova, Andrea Madarasova Geckova, Jitse P. van Dijk, Sijmen A. Reijneveld

**Affiliations:** 1 Department of Health Psychology and Research Methodology, Medical Faculty, P.J. Safarik University in Kosice, Trieda SNP 1, 040 11 Kosice, Slovakia; andrea.geckova@upjs.sk; 2 Olomouc University Society and Health Institute, Palacky University in Olomouc, Univerzitni 22, 771 11 Olomouc, Czech Republic; j.p.van.dijk@umcg.nl; 3 Department of Community and Occupational Medicine, University Medical Center Groningen, University of Groningen, Antonius Deusinglaan 1, 9713 AV Groningen, The Netherlands; s.a.reijneveld@umcg.nl

**Keywords:** deprivation, unemployment, employability, mechanisms, segregated Roma

## Abstract

Our earlier article showed that increased employability of segregated Roma may improve their well-being and health. To achieve that, appropriate employment based on a public–private partnership could be the key. For optimal design of such a partnership, we need insight into its potential mechanisms. Evidence on this is lacking, however. This paper builds on the previously published article by focusing on mechanisms for achieving better health. Therefore, our aim was to identify the potential mechanisms by which a public–private Roma employment project could increase employability. We investigated a Roma employment project called Equality of Opportunity established by a private company, U.S. Steel Kosice in eastern Slovakia. We conducted a multi-perspective qualitative study to obtain key stakeholders’ perspectives on the potential mechanisms of a public–private Roma employment project in terms of increased employability. We found three types of mechanisms. The first type regarded formal job mechanisms, such as an appropriate employment and salary offer and a bottom-up approach in capacity building. The second type involved sustainability mechanisms, such as the personal profile of project and work-shift coordinators, the continuous offer of training and cooperation with relevant stakeholders (municipalities, community centers, etc.). The third type was cultural mechanisms, such as personal contact with project participants, attention to less-voiced groups like children, the motivation of project participants, a counter-value reciprocity approach and respect for the specifics of Roma history. Our findings imply that policymakers could consider public–private partnerships for increasing the employability of segregated Roma, as they have the potential to address a wider range of social needs simultaneously.

## 1. Introduction

Roma are one of the largest ethnically delineated populations in Europe [1]. Substantial proportions of Roma reside in poor segregated communities. The poor health status of segregated Roma represents the most persistent health inequalities in Central and Eastern Europe (CEE) and is associated with a history of prejudice and discrimination, very low levels of education and income and high rates of unemployment, compared with the general population [2]. Reducing unemployment among segregated Roma has been defined as a key priority for improving their health outcomes [3]. Huge funds have been earmarked for this priority, but the effects seem to be minimal or at least questionable in most cases [4].

The low levels of employment of segregated Roma are related to both their suitability for the labor market and the structure of the labor market. Unemployment is increasingly understood to be caused by a lack of employability [5], which refers to a set of skills, knowledge, understanding and personal attributes that make a person more likely to gain and maintain employment or to obtain new employment, if required [6]. This is especially important, as poor employability is a key feature of segregated Roma [7]. However, the low competitiveness of segregated Roma is not the only barrier to employment. Better employability does not always increase their employment opportunities, because other factors, such as anti-Gypsyism, also affect their chances of getting a job [8,9,10]. Because of these discriminatory practices, Roma often have access only to unstable jobs with low wages [11]. The issue of segregated Roma is thus complex, and simple job creation seems to be an insufficient solution. This implies that a more comprehensive approach is needed that covers all the above-mentioned domains [12].

Public–private partnerships may be a way to improve the poor employment of segregated Roma. Public and private partners have been shown to separately lack sufficient capital to create a sustainable solution for reducing the unemployment of segregated Roma [3]. However, establishing a partnership between them may be the answer [8]. In the context of segregated Roma, we understand public–private partnership to be a platform for cooperation between the private sector (small and medium-size enterprises, large businesses), the public sector (the state, municipalities and schools) and ideally the third sector (non-governmental organizations—NGOs) with the aim of increasing the employability of segregated Roma. The private sector may help provide employment and training opportunities for Roma, whereas the public and third sector can be helpful in providing adequate potential employees to employers together with various types of support within the process. Such a partnership could have huge potential to increase employability by combining their assets, offering appropriate and equal work opportunities, initiating a dialogue within the business community regarding zero tolerance to discrimination [8] and developing and enforcing laws and workplace policies against discrimination.

To achieve this, we need insight into the mechanisms that lead to the employability of segregated Roma in order to design adequate public–private partnerships. Our previous article from the same study showed that a Roma employment project based on a public–private partnership may increase the employability of segregated Roma as well as improve their well-being and health [8]. In order to allow replication of this best practice, we should get insight into the mechanisms leading to its success, i.e., how and why such a project works [13]. The identification of mechanisms could then help to unpack the assumptions underlying the intervention, and this knowledge could be then used to better inform the design and evaluation of social policies and programs [13]. Evidence on these mechanisms is lacking, however, and multi-perspective comprehensive studies on such public–private partnerships that aimed to increase Roma employability are needed. Therefore, the aim of this paper was to identify the potential mechanisms by which a public–private Roma employment project could increase employability. This paper builds on our previously published article [8] by focusing on the mechanisms for achieving better health.

## 2. Materials and Methods

### 2.1. Theory and Hypotheses

This paper is the result of a large study on health inequalities under the project 7FP SOPHIE (Evaluating the impact of structural policies on health inequalities and their social determinants and fostering change), on which one paper has been published before [8]. Generally, in that study, we built on the public health theory of health determinants [14], postulating that not only do constitutional factors and individual lifestyle matter but also social and community networks as well as socioeconomic, cultural and environmental conditions that impact health. We further built on the theory of health inequalities [15], arguing that inequalities in health arise because of inequalities in society—in the conditions in which people are born, grow, live, work and age, and taking action to reduce health inequalities does not require a separate health agenda but action across the whole of society. Finally, we built on the theory of hard-to-employ groups [16], explaining that certain groups of individuals (also including minorities, typically sharing common characteristics of inadequate income, poor housing, inferior education, a lack of medical attention and lack of real job opportunities) tend to experience unusually high or prolonged levels of joblessness even in relatively good times, and their needs typically go beyond the scope of assistance available at traditional employment agencies and welfare offices.

Conceptually, we based our manuscript on a program theory for the project that we assessed (with a special focus on the mechanisms domain in this article), addressing the causal processes that link the implemented treatment to outcomes [13]. For this, we used the Context, Mechanism, Outcome (CMO) theory [17] as our conceptual framework based on what works, for whom and in what circumstances. We further considered a number of other theories which provided strong guidance for our research design (e.g., research questions, units of analysis), such as individual theories (individual perception, personality, interpersonal interactions, etc.), group theories (informal groups, work teams, interpersonal networks, etc.), organizational theories (organizational structure and functions, organizational partnerships, etc.), and social theories (functionalism, conflict theory, social constructivism, symbolic interactionism), etc.

### 2.2. Design

We conducted a multi-perspective qualitative study investigating a Roma employment project called Equality of Opportunity, established in 2002 by U.S. Steel Kosice (USS Kosice). This project represents, in terms of size, duration, complexity and sustainability, an interesting example of an attempt to address the Roma social inequality issue. We used the Context, Mechanism, Outcome (CMO) framework to structure data collection. The CMO configuration makes up part of the realist evaluation approach [17] and aims to deliver a proposition stating what works, for whom and in what circumstances. This may increase the understanding of the effectiveness of the program, with an explanation of why the outcomes developed as they did, how the project was able to act on the underlying mechanisms and in what contexts [18]. We will report only on the mechanisms in this paper.

The study consisted of several phases. The research protocol included a detailing of the data collection, i.e., the way to set up the project stages, procedures and timetable; the field procedures and data collection structure; and the questions, sample, report guide and sources of evidence. Details on the study stages and the structure of the interviews are provided in the Appendix. The second step was to collect data on the setting of the project (project background). The third step was to collect data using direct observation, in-depth semi-structured interviews, focus groups and informal face-to-face unstructured interviews. The last step was to analyze data in two rounds: the first round ran along with the data collection, and the second round was performed later. More detailed information on these stages can be found in Appendix A.

### 2.3. Sample

We included the main actors in the project, i.e., the Roma community, professionals (labor, education), public authorities and others (a priest, a nun and a cultural anthropologist), with proper methods of data collection for each group. The final sample consisted of 20 respondents (55% male) for the formal and informal interviews together, 28 respondents (39% male) for the focus groups and 98 respondents (gender not monitored) for direct observation (Table 1).

### 2.4. Procedure

We collected data using in-depth semi-structured interviews, informal unstructured interviews, focus groups and direct observation. We carried out the in-depth semi-structured interviews using a predefined set of topics and open-ended key questions, with the aim of systematically covering all topics of interest (mechanisms, with a special focus on increasing employability). We performed these interviews face-to-face, collecting the data by audio recording (with informed consent from participants) and by written field-notes. The layout and structure of the interviews are presented in Appendix B.

We carried out the informal unstructured interviews to gain the views of people not directly involved in but possibly affected by the project. These interviews were done by one researcher accompanied by a Roma community worker who, if necessary, also translated from and to the Romani language. The researcher collected the data using written field-notes.

We conducted focus groups using a predefined set of topics and open-ended key questions to gain the views of people not directly involved in but possibly affected by the project. We conducted three focus groups, each by three researchers, with the contents of interviews collected by written notes. The focus group with Roma children was facilitated by a Roma assistant who, if necessary, also translated from and to the Romani language. The layout and structure of the focus group scenario are presented in Appendix C.

Finally, we used non-participatory direct observation without interaction during the recruitment process to examine participants, their settings and their practices when applying for a job in the project. During this recruitment, we also observed the attitudes and habits of USS Kosice representatives. Furthermore, we directly visited the residences of participants in an effort to capture life in the settlements. For this, we used direct observation of participants with interaction. During the visits to the settlements, we were without USS Kosice representatives, accompanied only by local community workers who, if necessary, also translated from and to the Romani language.

### 2.5. Measures

We collected data on the setting of the project, the characteristics of the participants and on potential mechanisms by which the project could increase employability. Regarding the project setting, we collected data on the project launch, including its circumstances and key personnel, the project’s main goal, the type of contracts, financial remuneration and the characteristics of the project participants. Regarding these characteristics, we assessed gender, locality, age and education. Age structure, education level and average number of workers were calculated based on the overall number of participants since the start of the project in 2002. We further collected data using the CMO framework on potential mechanisms by which the project could increase employability. We understood these mechanisms to be measures (activities, tools, actions) that triggered specific outcomes within a particular context. This regards only mechanisms/measures from the CMO framework. Examples of questions regarding the mechanisms of the CMO framework are shown in Table 2.

### 2.6. Analysis and Reporting

First, we described the project setting (the project background). Second, we assessed the mechanisms potentially leading to increased employability of segregated Roma using the CMO framework. For this second step, we performed a content analysis of the data based on recurrent abstraction, i.e., repetition of reading and summarizing in steps of data coded as relating to the same topics or variables [19]. The content analysis was performed separately by three researchers with the aim of finding common themes (searching for themes, reviewing themes, defining and naming themes) in order to identify contexts, mechanisms and outcomes, as seen by stakeholders. Both written notes and the recordings, after transcription, were coded manually. The answers regarding the mechanisms were given sub-codes, as in a typical qualitative analysis. At the end, the results of the separate analyses by three researchers were compared, and differences were discussed and resolved. Lastly, the final version was discussed, agreed on and finalized. Labels of individual mechanisms and three types of mechanisms were determined following consensual discussions among the researchers.

## 3. Results

### 3.1. Project Setting

The study was conducted in the setting of the Equality of Opportunity project. This project was financed by USS Kosice and carried out in cooperation with the municipalities comprising the adjacent Roma settlements. USS Kosice, an integrated steel producer, is a subsidiary of the United States Steel Corporation headquartered in Pittsburgh, USA, and the largest private employer in eastern Slovakia. The project was initiated in 2002 by the first president of USS Kosice, who offered job vacancies in response to a request for support to reduce a number of problems (increasing levels of criminality and debts and a high level of unemployment) in the nearby Roma settlement. USS Kosice offered Roma jobs with a significantly higher salary than the minimum wage and with training, while municipalities covered the selection of the candidates. Successful candidates formally became employees of the municipality but were assigned to USS Kosice for temporary work. USS Kosice has created around 170 jobs for segregated Roma since 2002 (having 0 jobs of this kind before this initiative). More extensive information can be found in Bosakova (2018) [20].

Project participants were all males and came mainly from three settlements that were within a 15 km radius of the USS Kosice plant. Ages of participants ranged from 18 to 60 years, and nearly three quarters were aged 21–40 years. Most project participants had completed only primary school (Table 3).

### 3.2. Mechanisms Related to Increased Employability of Segregated Roma

**Figure 1:**
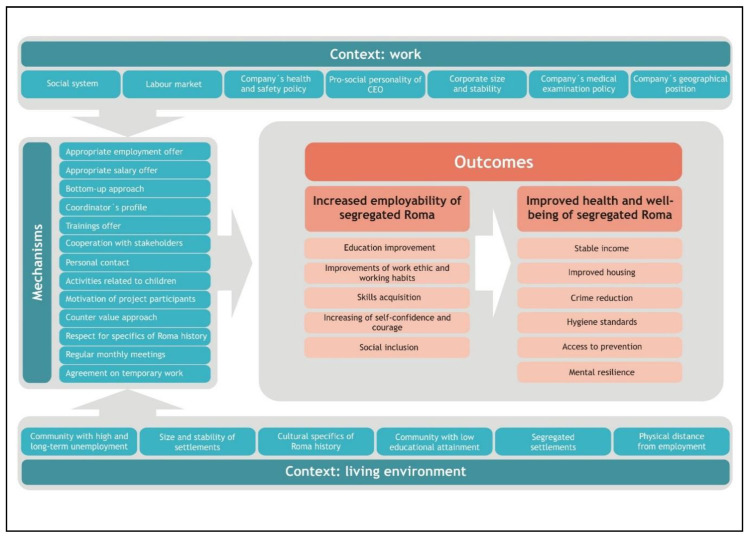
CMO framework of mechanisms leading to increased employability of segregated Roma.

Informants perceived three types of mechanisms that lead to an increase in the employability of segregated Roma. The first type concerned formal job mechanisms (Figure 1), with sub-mechanisms such as appropriate employment and salary offer and a bottom-up approach in capacity building. Table 4 provides examples of the narratives of the different interviewed agents and how they were obtained (e.g., focus groups, interviews, etc.) for the topics related to formal job mechanisms.

The second type of mechanism involved sustainability mechanisms (Figure 1), with sub-mechanisms such as the personal profile of project and work shift coordinators, the offer of continuous training and cooperation with relevant stakeholders (municipalities, community centers, other NGOs, etc.) Regular monthly meetings and agreement on temporary work assignments (between the municipality and company) were also mentioned but only by professionals and public authorities. Table 5 provides some examples from the narratives of the different agents interviewed and how they were obtained (e.g., focus groups, interviews, etc.) for the topics related to sustainability.

The third type of mechanism involved cultural mechanisms (Figure 1), with sub-mechanisms such as personal contact with project participants, attention for less-voiced groups (like children), motivation of project participants, the counter-value reciprocity approach, and respect for the specifics of Roma history. Table 6 provides some examples of the narratives of the different agents interviewed, and how they were obtained (e.g., focus groups, interviews, etc.) for the topics related to the cultural mechanisms that frequently arose during the data collection.

## 4. Discussion

We explored the potential mechanisms of a public–private Roma employment project in terms of increased employability. As mentioned in the Results, we found three types of mechanisms. The first type was formal job mechanisms, such as an appropriate employment and salary offer and a bottom-up approach in capacity building. The second type involved sustainability mechanisms, such as the personal profile of the project and work shift coordinators and the continuous offer of training and cooperation with the relevant stakeholders (municipalities, community centers, other NGOs, etc.) Regular monthly meetings and agreement on temporary work assignments (between municipality and company) were also mentioned but only by professionals and public authorities. The third type consisted of cultural mechanisms, such as personal contact with project participants, attention to less-voiced groups such as children, motivation of project participants, a counter-value reciprocity approach and respect for the specifics of Roma history. Stakeholders considered these three types of mechanisms to lead to an increase in the employability of segregated Roma. We will further discuss these results, i.e., the three main groups of mechanisms, in more detail.

### 4.1. Formal Job Mechanisms

Informants mentioned appropriate work and salary offer and a bottom-up approach to capacity building as the formal job mechanisms that led to the better employability of segregated Roma. In terms of increased employability, this group of mechanisms may help them gain work experience, acquire skills and improve knowledge and motivation to maintain employment. An appropriate work offer is aligned with the current capabilities of this workforce but does not have to be only menial [11], and together with bottom-up approach, it offers an opportunity to move forward [20]. A bottom-up approach allows gradual building of individual capacity and resilience and thus enables participants to learn new skills and access employment [12]. It presents a gradual step-by-step approach helpful in acquiring or refreshing working habits as well as gaining skills, qualifications and experience [21]. This approach also gives project participants enough time to adapt and become familiar with the rules of the majority and for project coordinators to map all participants and their abilities, giving them the opportunity to develop but also to understand the specifics of Roma history [20]. In regard to the salary offer, Roma often receive lower wages than non-Roma [11], which may discourage job-seeking and mostly motivate someone to remain on social benefits [22]. Public–private partnerships may help here to ensure sufficient, appropriate and equal opportunities for Roma by the entry of private capital (with a salary higher than the minimum wage or the sum of social benefits) on the private side and the monitoring of equality (with the salary equal to non-Roma) on the public side. In addition, decision-makers, when tackling the problem of hard-to-employ-groups such as segregated Roma, could consider a bottom-up approach enabling gradual capacity and resilience building.

### 4.2. Sustainability Mechanisms

Informants further mentioned a number of mechanisms, such as the personal profile of the project and work shift coordinators, the continuous offer of training and cooperation with relevant stakeholders (municipalities, schools, NGOs, community centers, etc.), which may contribute not only to the sustainability of the project but also to the sustainability of the participants’ involvement in the project. In terms of increased employability, this group of mechanisms can help develop personal attributes and understanding, improve knowledge and enhance skills also beyond work. The coordinator’s profile in terms of being a person with adequate capacity and skills is one of the critical success factors [12]. Musinka [23] also highlights a strong personality, assiduity and tenacity as some characteristics, which coordinators in this kind of project should have in order to be successful. Training helps to increase productivity and provides incentives to remain in employment [3]. Roma themselves often perceive the training offered by public institutions as inadequate and useless with regard to their chances on the labor market [4]. These include, for example, training in managerial skills, often regarded by Roma useless given their chances to access managerial positions. Thus, to be effective, the offer of training needs to be up to date and in line with the current labor market requirements for Roma [24,25], which could also be ensured by public–private partnerships. Such training could, for example, reflect the increasing importance of the Information and Communication Technologies (ICT) sector in the region and the high demand for ICT workers. This kind of training could further reflect shortages in the labor force of public services in the region, such as health care and social work services. Cooperation with stakeholders (municipalities, community centers, other NGOs, etc.) is important for sustainability [21]. It also strengthens a project’s capacity to address important issues beyond employment, mainly by connecting and combining the knowledge, capacities, experience, skills and networks of the individuals and institutions involved. Cooperation with municipalities, which often know all of their inhabitants personally, may help to recruit suitable Roma employees, improve ties with the community as a whole [3] and solve potential issues directly [20]. Cooperation with community centers and other NGOs may help with providing social services, together with mentoring, counselling and assistance to segregated Roma, their families and the whole community. These stakeholders can serve as a local partner and/or mediator between a company and segregated Roma employees. Moreover, Nasture [26] points out that non-existent or problematic cooperation among stakeholders is a significant barrier in Roma employment initiatives. Adequate prevention of and coping with such cooperation should be seriously considered when designing employment projects for segregated Roma.

Regular monthly meetings and agreement on temporary work assignments (between municipality and company) were also mentioned as promoting sustainability but only by professionals and public authorities. Regular monthly meetings provide coordinators room for evaluation, enable better monitoring of what works [12] and provide space for engaging participants, where their ideas can be freely presented and considered [20]. Agreements on temporary work assignments (between municipality and company) present a model of employee leasing, where employees are employed by the municipality but perform their work at a private company. The private company provides an employment opportunity and a salary but leaves administration to the public organization, which enables them to focus more deeply on the project and its participants. This also has huge potential in terms of engaging private companies to employ segregated Roma and consequent initiation of a much-needed dialogue within the business community regarding zero tolerance to discrimination and prejudice in the workplace [3]. However, the temporary character of this model may affect employees negatively; therefore, the possibilities of more stable work should also be considered. Regular monthly meetings could help increase segregated Roma engagement as well as help perform ongoing evaluation and follow-up improvements of the project. Agreements on a temporary work assignment (between municipality and company) could be an appropriate initial model for engaging private companies to employ segregated Roma.

### 4.3. Cultural Mechanisms

Informants further mentioned a number of mechanisms related to culture that could enhance the success of public–private partnerships in relation to the employability of segregated Roma, such as personal contact with project participants, attention for less-voiced groups like children, motivation of project participants and respect for the specifics of Roma history. In terms of increased employability, this group of mechanisms may help to develop personal attributes, improve knowledge and understanding and increase the motivation to gain and maintain employment.

Personal contact, as a mechanism that may increase the employability of segregated Roma, is crucial, when we consider the stigmatization of Roma outside the settlement and the often tense relationships between Roma and non-Roma [27]. This supports the so-called contact hypothesis, i.e., that personal contact reduces discrimination and stigmatization [28]. Personal contact enables the sharing of information, experiences and feelings and thus the forming of stronger ties outside the settlement [23]. This is important, as non-Roma typically lack information regarding most aspects of everyday life in a Roma settlement and believe that Roma are naturally unable to maintain non-Roma standards [27]. Non-Roma also often lack experience and information on the efforts that Roma have to make in regard to employment and the constraints they have to face [27]. Personal contact also enables an understanding of the wider family and community issues that Roma face and is crucial to developing trust between Roma and non-Roma—a key to success in this kind of project [12]. When operating an employment project for segregated Roma, decision-makers should emphasize this mechanism.

Informants also mentioned attention for less-voiced groups like children as a mechanism related to increasing the employability of segregated Roma. This mechanism is based on the life course way of thinking and includes close cooperation with local primary schools (financial and practical support) but also the involvement of children in various projects, attempting to motivate them to complete primary education and continue their studies at least at partner vocational schools [20]. This is crucial, as youth lacking appropriate means to complete their education and exposed to dominant self-exclusionary views and misinformation gradually resign [27] and most likely become adults who lack the means and motivation to continue their studies and/or become employed. Such activities may be part of all projects of this kind, as they include motivation and encouragement of children to acquire an education and skills that could increase their opportunities on the labor market in the future.

Motivation, a counter-value reciprocity approach and respect for the specifics of Roma history were seen as other mechanisms that may help to increase the employability of segregated Roma. Non-Roma often act towards the Roma in a discriminatory and racist manner, and even honest attempts to provide support to Roma, typically drawn on various misinformed concepts, are practically inapplicable [27]. Motivation in terms of encouraging, evaluating good results and appreciating is essential for increasing the employability of segregated Roma [23]. In addition, a counter-value approach in terms of reciprocity and supporting engaged attitudes seems to be important when tackling the employability of segregated Roma [20]. Specifics of Roma history have influenced the creation of several barriers, such as inter-generational poverty and the self-exclusionary ideologies of segregated Roma, together with different perception of values, which most likely have influenced the relationship of the community to employment and education [20,27] and led to their poor employability [7]. Therefore, trustful, inter-ethnic understanding and awareness of internal community complexities, past histories, culture, differences and aspirations are preconditions for establishing meaningful cooperation [12].

### 4.4. Added Theoretical Value

This study addressed the mechanisms by which a public–private Roma employment project could increase employability, departing from a constructed program theory [13] of the project concerned. Our findings generally confirm the line of thinking that was behind this project, i.e., that a public–private partnership leads to an appropriate work setting, which further leads to positive experiences at work, and these lead to better skills, resulting in improved chances for work and for obtaining better work, which then improves chances for better health. Our findings also contribute to the theory of hard-to-employ groups [16] and its following idea of using different strategies and greater public–private partnership involvement for employing hard-to-employ groups [29]. They can also further be explained based on the central theory that was the basis of the whole project, i.e., the theory of social determinants of health [14]. The study adds to that theory a detailing of the mechanisms by which these determinants act specifically for such a deprived group as Roma: formal job mechanisms, sustainability mechanisms and cultural mechanisms. It provides similar added value to the theory of health inequalities [15].

### 4.5. Strengths and Limitations

Our study has several strengths, the most important one being its wide range of informants and sources, which enabled various perspectives to be identified. This approach increased the robustness and transferability of the findings. However, some limitations need to be mentioned. We used a qualitative design for analyzing this particular best practice, which does not allow generalization to other communities. We think, however, that our findings represent a typical example of the issues related to highly deprived groups like Roma. Furthermore, social desirability may have affected the responses. However, we used a multi-informant strategy to get a full picture.

### 4.6. Implications for Practice, Policy and Research

The results of our study have implications for improving social policies. They imply that policymakers could consider public–private partnerships for increasing the employability of segregated Roma but with an emphasis on ensuring transparency to prevent any abuse. Such partnerships could provide gains in equal work and salary opportunities for all, regardless of ethnicity. Further, gradual capacity building via a bottom-up approach and training with respect to the specifics of Roma history may also be considered when designing similar projects. In addition, policy-makers should address a wider range of social needs simultaneously by reinforcing mutual cooperation with the relevant stakeholders, such as municipalities, community centers and other NGOs. Thus, they need to coordinate simultaneously across different fields covering not only employment but also education, housing, health and family, as the employability of individuals is often interrelated with other dimensions as well as with the employability of their family and wider communities, which need to be addressed as well.

A next step in future research may be to develop a longitudinal quantitative study to assess the actual impact of such a program over time, as well as the mediators of these effects and the degree to which various contextual factors moderate these associations. Future research could also assess more in-depth mechanisms on the employability of Roma in other regions or settings, which may yield large gains in the employment of this deprived group.

## 5. Conclusions

We conclude that potential mechanisms exist within a public–private Roma employment project that may help to increase employability of segregated Roma. The identified mechanisms are related to formal job mechanisms, such as appropriate and equal employment and salary opportunities, and gradual capacity building, which together could help segregated Roma gain work experience and skills and improve their knowledge and motivation to maintain employment. There are also sustainability mechanisms, such as the personal profile of the project and work shift coordinators, an offer of continuous training and cooperation with the relevant stakeholders (municipalities, schools, NGOs, community centers, etc.), which may help to develop personal attributes and understanding, improve knowledge and also develop skills other than working skills. Lastly, there are also cultural mechanisms, which may help segregated Roma develop their personal attributes and improve their knowledge and understanding and increase the motivation to gain and maintain employment. These relate to personal contact with participants, attention for less-voiced groups like children, motivation of participants, a counter-value reciprocity approach and respect for the specifics of Roma history. The identified mechanisms could help increase the employability of segregated Roma and thus their actual employment, which may help improve their living situation in a disadvantaged setting.

## Figures and Tables

**Table 1:** Characteristics of the samples participating in the various data collection methods.

Data Collection Method	Roma Community		Professionals		Public Authorities		Others	
	*n*	Description	*n*	Description	*n*	Description	*n*	Description
In-depth semi-structured interviews	3	Roma project participants	3	representatives of USS Kosice ^1^	3	officers from the City Council of Kosice	1	priest at Kosice-Lunik IX ^2^
	-	-	-	-	1	local authority of Velka Ida ^3^	1	cultural anthropologist
Informal unstructured interviews	2	wives of project participants	-	-	-	-	1	nun at Kosice-Lunik IX
	3	inhabitants from the segregated settlement in Velka Ida not participating in the project	-	-	-	-	-	-
	2	community workers in Velka Ida	-	-	-	-	-	-
Focus groups	17	Roma children from the elementary school in Velka Ida	5	teachers at the elementary school in Velka Ida	6	representatives/ workers at the Labor Office in Kosice	-	-
Direct observation	ca. 25	Roma job seekers during the recruitment process	3	representatives of USS Kosice during the recruitment process	-	-	-	-
	ca. 50	inhabitants of Velka Ida	-	-	-	-	-	-
	ca. 20	inhabitants of Kosice-Lunik IX	-	-	-	-	-	-

Note: ^1^ U.S. Steel Kosice; ^2^ Kosice-Lunik IX is a city district of Kosice close to USS Kosice, which is the largest Roma urban settlement in Slovakia; ^3^ Velka Ida is a village in the immediate vicinity of USS Kosice with a segregated Roma settlement.

**Table 2:** Examples of questions in the data collection based on the Context, Mechanism, Outcome (CMO) framework related to mechanisms.

Variable	Questions
Mechanisms	Which factors, elements and mechanisms have enabled the project implementation and maintenance (sustainability)? Which factors, elements and mechanisms have precluded and hindered the project implementation and maintenance? Which factors, elements and mechanisms within this project help to increase employability?

**Table 3:** Characteristics of the project participants (Equality of Opportunity, USS Kosice, Slovakia).

Characteristics *		Share (%)
Education	primary school	56%
	secondary school without graduation	18%
	secondary school with graduation	26%
Age structure	18–20	8%
	21–30	38%
	31–40	33%
	41–50	13%
	51–60	8%
Locality	Velka Ida ^1^	52%
	Kosice-Lunik IX ^2^	19%
	Kosice-Saca ^3^	29%

Note: * The average number of project participants per year was 111. ^1^ Velka Ida is a village in the immediate vicinity of USS Kosice with a segregated Roma settlement; ^2^ Kosice-Lunik IX is a city district of Kosice close to USS Kosice and is the biggest Roma urban settlement in Slovakia; ^3^ Kosice-Saca is a city district of Kosice with a Roma urban ghetto close to USS Kosice.

**Table 4:** Quotes illustrating findings related to formal job mechanisms that generated outcomes relating to the increased employability of segregated Roma, categorized by sub-mechanisms.

Quotes	Sub-Mechanism
*“Regarding the job category, participants perform work appropriate to their level of education attainment. These are at the beginning, in particular, auxiliary and cleaning jobs. But there is, of course, the possibility of getting more professional jobs, such as work in the Divisions* [divisions of the USS Kosice]. … *Promotion to a skilled and better-paid position depends above all on the willingness to learn, on a consistently good performance and on safety at work.”* (Representative of USS Kosice, in-depth semi-structured interview)	Appropriate employment and salary offer
*“They are not sitting at home, where they would regress more. … Of course it is not a position with huge intellectual growth. It offers rather human growth and the growth of positive habits. The project lifts them* [project participants] *out of misery. It gives them* [project participants] *value and freedom. That is why many* [project participants] *like to work there* [in the project] *…dignity and self-worth. There are also many* [project participants] *who leave for a better job elsewhere, and that is ok, it is great! This job* [in the project] *helped them to grow and to be successful on the labor market.”* (Priest and Nun at Kosice-Lunik IX, in-depth semi-structured interviews)	
*“What is important here is that the basic salary is already significantly higher in the first skill category than the minimum wage, so they* [project participants] *are motivated to work in the project. … Moreover, they also get a thirteenth and fourteenth* [monthly] *salary and various bonuses.”* (Officer from the City Council of Kosice, in-depth semi-structured interview)	
*“The financial remuneration* [of project participants] *has been gradually increased over the years. Now* [at the time of the interview], *their* [project participants] *gross wage is more than 30% higher than at the beginning of the project in 2002. Wages also rise, by a further 15% after completion of the training* [Operation of belt conveyors]. *Project participants are also entitled to the relevant bonuses, for example, for difficult working conditions* [shift work, etc.] *or as a result of a positive monthly evaluation. A further increase* [in a project participant’s wage] *is similar as with permanent employees, under the collective agreement.”* (Representative of USS Kosice, in-depth semi-structured interview)	
*“We are getting better; we live better* [than those who do not work in the project]. *We can afford more.”* (Wife of a project participant, Informal unstructured interview)	
*“Everything is done gradually. The selection process consists of five bottom-up phases. Advancement, when shifting to the higher skills category or from temporary to permanent employment, is also gradual.”* (Representative of USS Kosice, in-depth semi-structured interview)	Bottom-up approach in capacity building
*“The system* [labor market in general; work opportunities for segregated Roma in general] *is set so that they* [government; other companies] *throw you into the water and you’re supposed to swim* [even if you can’t]. *Here* [USS Kosice project] *they* [USS Kosice] *will teach you to swim first.”* (Community worker, Informal unstructured interview)	
*“It* [bottom-up capacity building] *provides sufficient time and space to adapt for both participants and coordinators.”* (Officer from the City Council of Kosice, in-depth semi-structured interview)	
*“It* [bottom-up capacity building] *ensures adequate time and space. Roma* [project participants] *have time to become familiar with the majority rules and also time to increase their skills and qualifications. And coordinators have time to understand them* [project participants], *create or modify some rules and also to map all the participant and their abilities and to give those who are the most reliable the opportunity to grow.”* (Representative of USS Kosice, in-depth semi-structured interview)	
*“The process of getting a job in the project is not easy. If the candidates are successful and also take part in some training, their chances of succeeding on the labor market grows rapidly.”* (Representative of USS Kosice, in-depth semi-structured interview)	

**Table 5:** Quotes illustrating findings related to the sustainability mechanisms that generated outcomes relating to increased employability of segregated Roma, categorized by sub-mechanisms.

Quotes	Sub-Mechanism
*“Foremen, as well as other coordinators who work with participants on a daily basis, perform a really admirable activity. They expend an enormous effort every day, because this work is not at all easy. It seems that for them this is not only a job, but a mission.”* (Local authority of Velka Ida, in-depth semi-structured interview)	Personal profile of project and work shift coordinator
*“Not just anyone can be involved in such work. The success of such a project always depends on coordinators who are patient, manful and who want to change something in the society.”* (Representative of USS Kosice, in-depth semi-structured interview)	
*“I think that good collaboration and communication between USS Kosice, the municipalities and the City Council leads to 50% of the project success”* (Officer from the City Council of Kosice, in-depth semi-structured interview)	Cooperation with various stakeholders
*“The company* [USS Kosice] *was involved in the establishment of the community center in Velka Ida and has cooperated with the community centers in Kosice-Lunik IX and Kosice-Saca …. We* [USS Kosice] *collaborated with the non-profit organization ETP Slovakia on various projects* [Chance for the Roma; Community on the way to prosperity] *aiming to increase the education of Roma children, young people and adults, with the main target group being project participants and their families.”* (Representative of USS Kosice, in-depth semi-structured interview)	
*“They* [USS Kosice] *closely collaborate with the local schools, involve children in various projects and so on. In the community center* [in Velka Ida, co-established by the USS Kosice], *various training, workshops and courses are carried out.”* (Community worker, Informal unstructured interview)	
*“…as already mentioned, one of the project objectives is also to upgrade the skills of participants; therefore, they* [project participants] *have the opportunity to regularly attend various kinds of training related to but also not directly related to their work.”* (Representative of USS Kosice, in-depth semi-structured interview)	Training
*“The training offered by the Labor Office is not helpful. The training in the project is at least practical*, *for example, for another job, too.”* (Wife of a project participant, Informal unstructured interview)	
*“Roma from the settlements do not have* [as unemployed persons] *any chance to gain or to improve their qualifications. Even the training offered by the Labor Office is inadequate and impractical. The project therefore has a substantial impact on improving their* [project participants] *skills and qualifications* [via offer and support of attendance in various kinds of training].” (Local authority of Velka Ida, in-depth semi-structured interview)	
*“The process of getting a job in the project is not easy. If the candidates are successful and also take part in some training, their* [project participants] *chances of succeeding on the labor market grows rapidly.”* (Representative of USS Kosice, in-depth semi-structured interview)	

**Table 6:** Quotes illustrating findings on the cultural mechanisms that generated outcomes related to increased employability of segregated Roma, categorized by sub-mechanisms.

Quotes	Sub-Mechanism
*“For them* [project participants], *a serious problem* [related to work attendance] *is that they have many children* [more than non-Roma]. *You have to realize it because, you know,* … *I didn’t have* [kids] *back then, now I do, and back then I didn’t* [realize, understand] … *but* … *what about with all those kids, right?* … *and when they have 10, sometimes 12 children and always* [often] *have to be at home, or take them to the doctor*… *so I said* [back then]: *“Why you? Why the father? Why not the mother?”* … *So he* [project participant] *said: “We have 10 kids, so where should I leave them?”* … *It’s not like that, just like with the whites* [non-Roma], *that if they don’t go to work, or whatever, we can simply terminate* [employment] … *it is not possible here, because if we had this set up, you know, exactly the same, you are not at work* [often], *you are fired, maybe we wouldn’t sit here today and the project wouldn’t exist anymore.*” [Researcher: So you have a little more benevolent approach?] *Yes* … *we have to* … *yes* … *we have to be* [more benevolent] *because otherwise it wouldn’t work.*” (Local authority of Velka Ida, in-depth semi-structured interview)	Respect for the specifics of Roma history
*“Participants* [project participants] *often have to stay at home and help with children or go to the doctor with some of them* [children], *while the mother takes care of the rest of them at home. It is a complication, but we have to understand.”* (Representative of USS Kosice, in-depth semi-structured interview)	
*“Most of the participants do not think conceptually, but rather impulsively, which often leads them to leave the job because of trifles. They* [project participants], *however, almost always come back. And we give them another chance.”* (Representative of USS Kosice, in-depth semi-structured interview)	
*“The Roma react impulsively. There is often nothing behind it, just momentary dissatisfaction or confusion. Regarding the job, oftentimes, they find something displeasing and leave, but then they almost always return. It is important to understand that this is not the mentality or lack of capacity, it is the way they have seen from childhood people around them face up to inner conflicts.”* (Cultural anthropologist, in-depth semi-structured interview)	
*“Some of them* [Roma] *come and work* [become a project participant], *some come, work for a while* [are in the project] *and leave, for example to England, and then come back and ask to work in the project again. And they* [USS Kosice] *allow them* [former project participants] *again*.” (Wife of a project participant, Informal unstructured interview)	
*“…I have motivation there* [USS Kosice project]. *I want to join the core staff one day in the future. I see they* [core staff] *have better salaries there, you know* [smile]. *So that is the better motivation there, you know* [smile]. *But I don’t complain, it is good, still better than a material need benefit of 60 Euros* [material needs benefit payment provided by the Office of Labor, Social Affairs and Family for unemployed or low-income families].” (Project participant, in-depth semi-structured interview)	Motivation of project participants
*“We selected each year the three best participants* [according to attendance, performance and adherence to safety rules] *and those joined the core staff. This dragged them* [project participants] *to work. To join the core staff was the strongest motivation for them* [project participants]. *They* [project participants] *wanted it very much, because they* [project participants] *knew that if they were core staff, they would be much better-off, because core staff earn huge money. They* [project participants] *were still waiting at the end of December to see who will be picked and who will join the core staff in January … they wanted this very much … they had a goal … when it was interrupted* [the possibility of joining the core staff was temporarily suspended in 2008 due to the economic crisis, when USS Kosice did not create any new jobs even outside the project], *they lost their motivation.”* (Local authority of Velka Ida, in-depth semi-structured interview)	
*“The introduction of a remuneration for attendance, safety and performance led to huge motivation. It helped a lot. Attendance improved, performance improved and adherence to safety did so as well.”* (Representative of USS Kosice, in-depth semi-structured interview)	
*“Non-financial motivation is also widely used. For example, organization of social and cultural events, in which project participants, their children and families are involved together with representatives of USS Kosice. These events have a huge success and seem to have even a considerable motivational and integrative character.”* (Representative of USS Kosice, in-depth semi-structured interview)	

## Appendix A. Stages of the Performed Qualitative Study

**Table A1:** Stages of the Performed Qualitative Study.

Phase	Description		Timetable
1. Study protocol	elaboration of study protocol: study objectives, timetable set-up, field collection procedures, data collection procedures, analytic strategy		January 2013
2. Preparation for data collection	specifying sites to be visited, data collection plan, contacting respondents		February–March 2013
3. Data collection	collecting and studying of internal documents and previous studies (relevant academic and grey literature)		March–September 2013 January–October 2015 June–December 2016 January 2017–February 2018 March 2018–May 2019
	direct observation	presence in recruitment process	March 2013
		visit to settlements (Velka Ida, Lunik IX)	April; June; November 2013 January; September 2014 December 2017 March 2018
	in-depth face-to-face semi-structured interviews		April–July 2013
	focus groups		April–July 2013
	face-to-face unstructured interviews		November 2013
4. Data processing/analysis	content analysis: transcription, coding, recursive abstraction (searching for themes, reviewing themes, defining and naming themes), writing and finalizing		July 2013–January 2014 June–October 2015 June–December 2016 January 2017–February 2018 March 2018–May 2019

## Appendix B. Structure of the Semi-Structured Interviews

1. Project initiation—human resources, financial assurance, social situation, legislation, material and technical support, locality….

(a) What are the circumstances of the project initiation? Why do you think this project was created? (physical neighborhood, high crime, high unemployment rate, available and cheap labor force…) (*Why and how was the project initiated?*)
(b) Which factors, elements and mechanisms (tools, instruments) affected the project start-up in a positive and a negative way? (legislation, local authorities, financial assurance, material and technical support….) (*Why and how has the project initiation been supported? Why and how has the project initiation been limited and restricted?*)

2. Project implementation and maintenance—human resources, financial assurance, social situations, legislation, material and technical support, locality…

(a) Which factors, elements and mechanisms (tools, instruments) have enabled project implementation and maintenance (sustainability)? (*Why and how was the project implemented? Why and how has the project been maintained?*)
(b) Which factors, elements and mechanisms (tools, instruments) have precluded and hindered project implementation and maintenance? (Legislative—existing social support scheme, Labor Code, fluctuation…) (*Why and how has project implementation and maintenance been supported? Why and how has project implementation and maintenance been limited and restricted?*)

3. Project impact and outcomes

(a) What do you think: what are the (positive and negative) outcomes of this project?
(b) What do you think: does the project have an impact on the rest of the community? If yes, why and how? If not, why?
(c) What do you think: does the project have an impact on increasing employability? If yes, why and how? If not, why? (Which factors, elements and mechanisms within this project help to increase employability?)

4. Well-being and health (health equity)

(*Why and how has the project affected health (health equity)? How may the project affect health (health equity)?*)

(a) Do you think the project improves the chances of participants, their families and children to be healthier? If yes, why and how? If not, why?
(b) Are the project participants in better shape than those from their surroundings who are not involved in the project? (Those employed and unemployed who are treated only by the existing health care system etc.) If yes, why? If not, why?
(c) Do the project participants have better health and living conditions than those from their surroundings who are not participating in the project? (Those employed and unemployed who are treated only by the existing health care system, etc.) If yes, why? If not, why?

**Specific investigator’s questions to keep in mind during the data collection!**

*What is the context of the project initiation? What is the context and what are the elements and mechanisms that enabled the successful implementation of the project and its maintenance?*

*What is the context and what are the elements and mechanisms that precluded and hindered the successful implementation of the project? (Barriers and limits of project initiation, implementation and maintenance).*

*What is the connection between the project and health equity? (Does the project have the impact on health equity?)*

## Appendix C. Scenario of the Focus Group Interviews

1. Presentation of the researchers,

2. Presentation of the study focus,

3. Short presentation of the participants

4. Questions:

(a) Project creation
  - Why do you think this project was created? (What are the circumstances of the project initiation?)
  - Do you know how this project was created?
  - Were there any obstacles at the beginning? (Which factors, elements and mechanisms affected the project start-up in a negative way?)
  - Was there any support at the beginning? (Which factors, elements and mechanisms affected the project start-up in a positive way?)
(b) Project now
  - Are there any obstacles now? (Which factors, elements and mechanisms preclude and hinder the project maintenance?)
  - Who or what presents the main support of the project? (Which factors, elements and mechanisms enable and support the project maintenance?)
(c) Project outcomes
  - What do you think: what are the (positive and negative) outcomes of this project? (What are the positives about this project? What are the negatives about this project?)
  - What do you think: does the project have an impact on the rest of the community? If yes, why? If not, why?
  - What do you think: does the project have an impact on increasing employability? If yes, why and how? If not, why? (Which factors, elements and mechanisms within this project help to increase employability?)
(d) Project versus well-being and health
  - Do you think the project improves the chances of participants, their families and children to be healthier? If yes, why and how? If not, why?
  - Are the project participants in better shape than those from their surroundings who are not involved in the project? (Those employed and unemployed who are treated only by the existing health care system etc.) If yes, why? If not, why?
  - Do the project participants have better health and living conditions than those from their surroundings who are not involved in the project? (Those employed and unemployed who are treated only by existing health care system etc.) If yes, why? If not, why?
